# Myopathy Associated with Treatment of Graves’ Disease

**DOI:** 10.3390/medicina57101016

**Published:** 2021-09-25

**Authors:** Yoon-Kyung Ji, Shin-Hee Kim

**Affiliations:** Department of Pediatrics, Incheon St. Mary’s Hospital, College of Medicine, The Catholic University of Korea, Incheon 21431, Korea; ppjyk00@naver.com

**Keywords:** graves’ disease, methimazole, myopathy, creatine kinase

## Abstract

Here, we report a case of an increase in serum creatine kinase (CK) concentration in an 11-year-old girl being treated for Graves’ disease with antithyroid drugs (ATDs). The patient complained of myalgia two weeks after methimazole treatment. Triiodothyronine (T3) and free thyroxine (FT4) levels were normal, but the serum CK level was significantly elevated. After switching to propylthiouracil, the serum CK level decreased to normal, and the myalgia was resolved. The development of myopathy during the treatment of hyperthyroidism may be considered as an adverse reaction of MMI. In this report, we present a rare pediatric case, along with a discussion on the possible causes of myopathy that occurred during the treatment of Graves’ disease. A careful follow-up (serum CK levels and thyroid function) and treatment reassessment should always be considered after antithyroid treatment.

## 1. Introduction

Thyroid hormone signaling regulates crucial biological functions, including energy expenditure, thermogenesis, development, and growth. Among other targets, skeletal muscle is a major target of thyroid hormone signaling [1]. Therefore, musculoskeletal symptoms are occasionally observed during thyroid hormone dysfunction [2]. Hypothyroid-induced myopathy typically presents with myalgia and CK elevation [2]. Similarly, patients with Graves’ disease usually develop proximal muscle weakness. Graves’ disease has been associated with autoimmune disorders of muscles, such as polymyositis and myasthenia gravies [3]. However, hyperthyroid-induced myopathy is rarely associated with elevated CK.

Previous studies reported that some Graves’ disease patients with oral antithyroid drugs (ATDs) manifested myalgia and increased serum CK levels [4,5,6,7]. Many patients reported taking ATDs, which were presumed to be the cause of muscular lesions [8,9]. We report a case of a child with Graves’ disease whose treatment with methimazole was complicated by myopathy with CK elevation.

## 2. Case Report

An 11-year-old girl was admitted to the outpatient clinic with goiter, weight loss, palpitations, heat intolerance, and sweating over a period of three months. According to clinical symptoms and laboratory results, the patient was diagnosed with Graves’ disease. The patient took 30 mg of methimazole (MMI) and 10 mg of propranolol per day, which improved most of her symptoms. After about one month of treatment, serum thyroid hormone values were in the normal range, the MMI dose was reduced to 15 mg per day, and the propranolol was stopped. However, two weeks later, she complained of myalgia of the neck, both shoulders, and proximal limbs and swelling in both thighs. She was referred to Incheon St Mary’s Hospital for further evaluation.

**Figure 1:**
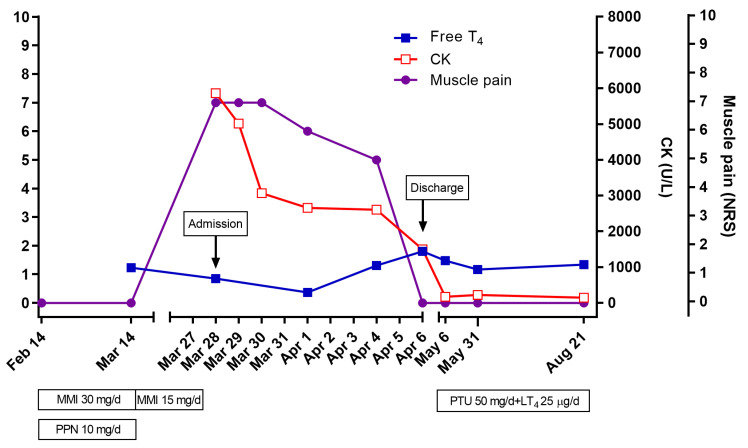
The clinical course of myopathy associated with treatment of Graves’ disease. CK, creatine kinase; LT_4_, levothyroxine; MMI, methimazole; NRS, numerical rating scale.

The systolic and diastolic blood pressure measurements were 100 and 60 mmHg at admission, respectively. The heart rate was recorded at 65/min beats/min and body temperature was 36.4 °C. The thyroid was enlarged diffusely without nodules. Laboratory findings revealed serum TSH, total T3, and free T4 levels were 0.03 μIU/mL (normal range 0.5–5.5 μIU/mL), 86.08 ng/dL (normal range 80–210 ng/dL), and 0.85 ng/dL (normal range 0.8–2.3 ng/dL), respectively. The TSH receptor antibody level was 36 IU/L (normal < 1 U/L). The serum CK level was 5865 U/L (normal range 0–250 U/L) and was elevated during symptom presentation. The serum lactate dehydrogenase (LDH) level was 705 U/L (normal range 200–450 U/L), and the aspartate aminotransferase (AST) level was 50 U/Lm (normal range 9–40), both slightly elevated. Additional laboratory tests were performed to rule out causes of autoimmune disease, including FANA, anti-histone Ab, anti-Sm Ab, and anti-Ro/La Ab, which were all within the normal ranges. Further investigations found normal serum aldolase and myoglobin and unremarkable EMG (electromyography)/NCS (nerve conduction study) results. Muscle biopsy was not performed. Ten days after MMI discontinuation, CK decreased to 1500 IU/L, and the muscle symptoms recovered. Because thyroid function tests showed a relapse of hyperthyroidism, the patient started taking 50 mg of propylthiouracil (PTU) instead of MMI, and l-thyroxine (25 mcg) was added (Figure 1). Four weeks later, the PTU was exchanged for MMI and l-thyroxine was added, the serum CPK level decreased to normal, and the muscle symptoms resolved. At that time, the levels of serum TSH, total T3, and FT4 were 0.01 μIU/mL, 207.64 ng/dL, and 1.48 ng/dL, respectively.

## 3. Discussions

Thyroid hormones play a central role in muscle function, and thyroid dysfunction can cause extensive myopathy [1]. The clinical symptoms of thyrotoxic myopathy are characterized by progressive weakness in proximal muscles, atrophy, and wasting, with a normal or decreased CK level [2]. Conversely, hypothyroid myopathy is a pathological change in the muscle–skeletal fibers and/or muscle damage with an increased CK level [10]. However, there are several cases of elevated serum CK levels, even in the absence of a hypothyroid condition during treatment for Graves’ disease [4,5,6,7]. Suzuki et al. reported the first cases of elevated CK levels and myopathy during treatment for hyperthyroidism [4]. Four adult patients with Graves’ disease developed an elevated CK level after MMI use, and three of them had musculoskeletal symptoms. Regardless of MMI reduction, the CK level decreased to within the normal range after the addition of l-thyroxine [4]. Mizuno et al. reported two cases of elevated CK in children treated with ATD for Graves’ disease, with the CK value elevated to a very high level (up to 11,630 IU/L) in one case [11].

Our case describes a similar clinical course. Our patient’s thyroid function returned to the euthyroid state six weeks after the initiation of MMI, but two weeks later, myalgia developed and the CK level increased. After changing the MMI to PTU and adding l-thyroxine to the treatment regimen, the CK level decreased to within the normal range and the myalgia was resolved.

Although the mechanism of this phenomenon is unclear, there are several possible explanations for myopathy that occurs during the treatment of Graves’ disease. The development of myopathy during the treatment of hyperthyroidism with ATDs may be considered an adverse reaction of ATDs [8,12]. There are a number of mechanisms, including direct effects of ATDs on myocytes, and immune-related responses secondary to ATDs [4,13]. These mechanisms rationalize the effects of reducing or stopping ATDs [4]. Given that the myopathy in the present case developed after the patient started receiving MMI, and the symptoms resolved after discontinuing MMI with the early switch to PTU, we conclude that the symptoms were induced by the MMI treatment. A recent case showed that a patient with Graves’ disease experienced fever, rash, and arthralgia after a 15-day treatment of MMI [14]. Upon stopping the drug treatment, the patient’s symptoms promptly disappeared [14]. Antithyroid arthritis syndrome is a constellation of symptoms of myalgia, arthralgia, arthritis, fever, and rash associated with the use of ATDs. It usually resolves without any sequela within several weeks after the cessation of the causative drugs [15].

Graves’ disease is associated with many muscular diseases, such as myopathy, hypokalemic paralysis, and myasthenia gravis. In this case, thyrotoxic hypokalemic periodic paralysis (THPP) and autoimmune-associated inflammatory myopathy were excluded due to normal potassium, FANA, anti-histone Ab, anti-Sm Ab, and anti-Ro/La Ab. We could not exclude the possibility of unresolved thyrotoxicosis as the cause of elevation in CK levels due to the lack of data on CK levels before MMI treatment. Although thyrotoxic myopathy is usually associated with normal CK levels, acute thyrotoxic myopathy can present with more severe muscle weakness with marked elevations of CK levels [16,17,18]. The symptoms and CK elevations in thyrotoxic myopathy could be resolved with the continuation of ATD and addition of analgesics, but this approach was not attempted in the present case. Laboratory abnormalities associated with hyperthyroidism, such as neutropenia and elevated transaminase, are usually normalized with ATDs, and they should not be regarded as contraindications to use ATDs [19,20].

In some literature, a rapid decrease in thyroid hormone levels was considered the cause for these myopathic changes because the onset timing of these changes was soon after the reduction in thyroid hormone levels [4,6]. Within the one to two months of ATD initiation, there was an onset of myalgia with a concurrent increase in CK and decrease in free T4 levels compared with initial values [7,8]. Lu et al. hypothesized that intracellular T3 equilibrium in skeletal muscles indicates adaptation by altering the expression or activity of related receptors and enzymes in the hyperthyroid state. A rapid decrease in thyroid hormone could result in muscle injury as in hypothyroidism. Serum CK can increase due to muscle fiber destruction and reduced CK clearance [1,7]. Myopathy that occurred after radioactive iodine therapy or thyroidectomy could not explain the ATD effect [10].

Our case supports the idea of myopathy associated with an adverse reaction due to MMI. Knowing the potential adverse effects associated with treatment for Graves’ disease can facilitate the proper management of the patient. Therefore, it is necessary to measure the serum CK level when symptoms of myalgia appear during the treatment of Graves’ disease. For patients with newly onset myopathy during treatment, it is important to adjust the ATD dose or replace it with another drug if an adverse reaction due to ATD is suspected.

## Data Availability

The data presented in this study are available on request from the corresponding author.

## References

[1] Salvatore D., Simonides W.S., Dentice M., Zavacki A.M., Larsen P.R. (2014). Thyroid hormones and skeletal muscle—New insights and potential implications. Nat. Rev. Endocrinol..

[2] Cakir M., Samanci N., Balci N., Balci M.K. (2003). Musculoskeletal manifestations in patients with thyroid disease. Clin. Endocrinol..

[3] Wang H., Li H., Kai C., Deng J. (2010). Polymyositis associated with hypothyroidism or hyperthyroidism: Two cases and review of the literature. Clin. Rheumatol..

[4] Suzuki S. (1997). Elevation of serum creatine kinase during treatment with antithyroid drugs in patients with hyperthyroidism due to Graves disease. A novel side effect of antithyroid drugs. Arch. Intern. Med..

[5] Kim H., Kim J., Huh R., Cho S.Y., Jin D.-K. (2015). Elevation of serum creatine kinase during methimazole treatment of Graves disease in a 13-year-old girl and a literature review of similar cases. Ann. Pediatr. Endocrinol. Metab..

[6] Li Q., Liu Y., Zhang Q., Tian H., Li J., Li S. (2017). Myopathy in hyperthyroidism as a consequence of rapid reduction of thyroid hormone. Medicine.

[7] Lu R., Wang H., Hong T., Gao H. (2020). Myopathy after rapid correction of hyperthyroidism. Medicine.

[8] Tsang C.C., Hui W.S., Lo K.M., Yeung J.H.M., Cheng Y.L. (2014). Anti-Thyroid Drugs-Related Myopathy: Is Carbimazole the Real Culprit?. Int. J. Endocrinol. Metab..

[9] Khalil R.B., Salbi M.A., Sissi S., El Kara N., Azar E., Khoury M., Abdallah G., Hreiki J., Farhat S. (2013). Methimazole-induced myositis: A case report and review of the literature. Endocrinol. Diabetes Metab. Case Rep..

[10] Shaheen D., Kim C.S. (2009). Myositis Associated with the Decline of Thyroid Hormone Levels in Thyrotoxicosis: A Syndrome?. Thyroid.

[11] Mizuno H., Sugiyama Y., Nishi Y., Ueda N., Ohro Y., Togari H. (2007). Elevation of serum creatine kinase in response to medical treatment of Graves’ disease in children. Acta Paediatr..

[12] Page S., Nussey S. (1989). Myositis in association with carbimazole therapy. Lancet.

[13] Lim A.Y.Y., Kek P.C., Soh A.W.E. (2013). Carbimazole-induced myositis in the treatment of Graves’ disease: A complication in genetically susceptible individuals?. Singap. Med. J..

[14] Xu M., Hou L., Chen M., Deng D. (2021). Antithyroid arthritis syndrome: A case report and review of the literature. Int. J. Clin. Pharmacol. Ther..

[15] Shabtai R., Shapiro M.S., Orenstein D., Taragan R., Shenkman L. (1984). The antithyroid arthritis syndrome reviewed. Arthritis Rheum..

[16] Lichtstein D.M., Arteaga R.B. (2006). Rhabdomyolysis Associated with Hyperthyroidism. Am. J. Med. Sci..

[17] Alshanti M., Eledrisi M.S., Jones E. (2001). Rhabdomyolysis associated with hyperthyroidism. Am. J. Emerg. Med..

[18] Hampton L.M., Farley M.M., Schaffner W., Thomas A., Reingold A., Harrison L.H., Lynfield R., Bennett N.M., Petit S., Gershman K. (2011). Prevention of Antibiotic-Nonsusceptible Streptococcus pneumoniae with Conjugate Vaccines. J. Infect. Dis..

[19] Scappaticcio L., Maiorino M.I., Maio A., Esposito K., Bellastella G. (2021). Neutropenia in patients with hyperthyroidism: Systematic review and meta-analysis. Clin. Endocrinol..

[20] Scappaticcio L., Longo M., Maiorino M.I., Pernice V., Caruso P., Esposito K., Bellastella G. (2021). Abnormal Liver Blood Tests in Patients with Hyperthyroidism: Systematic Review and Meta-Analysis. Thyroid.

